# Effects of neonatal fentanyl on late adolescent opioid-mediated behavior

**DOI:** 10.3389/fnins.2023.1094241

**Published:** 2023-02-14

**Authors:** Cynthia A. Crawford, Jordan A. Taylor, Ginny I. Park, Jasmine W. Rios, Joseph Bunch, Constance J. Greenwood, David Y. Lopez Sanchez, Diego J. Gonzales

**Affiliations:** Department of Psychology, California State University, San Bernardino, San Bernardino, CA, United States

**Keywords:** fentanyl, self-administration, antinociception, opioid, ontogeny

## Abstract

**Introduction:**

Because of the steady increase in the use of synthetic opioids in women of childbearing age, a large number of children are at risk of exposure to these drugs prenatally or postnatally through breast milk. While there is older literature looking at the effects of morphine and heroin, there are relatively few studies looking at the long-term effects of high-potency synthetic opioid compounds like fentanyl. Thus, in the present study, we assessed whether brief exposure to fentanyl in male and female rat pups during a period roughly equivalent to the third trimester of CNS development altered adolescent oral fentanyl self-administration and opioid-mediated thermal antinociception.

**Methods:**

We treated the rats with fentanyl (0, 10, or 100 μg/kg sc) from postnatal day (PD) 4 to PD 9. The fentanyl was administered daily in two injections given 6 h apart. After the last injection on PD 9, the rat pups were left alone until either PD 40 where they began fentanyl self-administration training or PD 60 where they were tested for morphine- (0, 1.25, 2.5, 5, or 10 mg/kg) or U50,488- (0, 2.5, 5, 10, or 20 mg/kg) induced thermal antinociception.

**Results:**

In the self-administration study, we found that female rats had more active nose pokes than male rats when receiving a fentanyl reward but not sucrose alone solution. Early neonatal fentanyl exposure did not significantly alter fentanyl intake or nose-poke response. In contrast, early fentanyl exposure did alter thermal antinociception in both male and female rats. Specifically, fentanyl (10 μg/kg) pre-treatment increased baseline paw-lick latencies, and the higher dose of fentanyl (100 μg/kg) reduced morphine-induced paw-lick latencies. Fentanyl pre-treatment did not alter U50,488-mediated thermal antinociception.

**Conclusions:**

Although our exposure model is not reflective of typical human fentanyl use during pregnancy, our study does illustrate that even brief exposure to fentanyl during early development can have long-lasting effects on mu-opioid-mediated behavior. Moreover, our data suggest that females may be more susceptible to fentanyl abuse than males.

## Introduction

Opioid use has reached epidemic levels in the United States. Over a 20-year period (1999–2011) in the United States, the annual number of opioid painkiller prescriptions rose precipitously, and in 2009, drug overdose deaths exceeded those from car accidents (Gardner et al., 2022). There has been a 200% increase in opioid overdose (poisoning) deaths since 2000, and 61% of all drug overdose deaths now involve opioids (Rudd et al., 2016). Drug overdose rates increased the most for persons aged 25–34 years, but there has been a sharp increase in the number of opioid overdoses in adolescents and young adults (15–24 years) in recent years. The synthetic opioid fentanyl has been particularly problematic with an 88% increase in fentanyl and fentanyl analog overdose deaths each year from 2013 to 2016 (Spencer et al., 2019; Han et al., 2022; Skolnick, 2022).

An unfortunate consequence of this crisis has been the increase in opioid use in women of childbearing age (Krans and Patrick, 2016; Hurley et al., 2020). Between 2008 and 2012, insurance records showed that roughly 30% of women in the childbearing age group filled a prescription for an opioid and the majority of people seeking treatment for opioid addiction are women (Yazdy et al., 2015; Krans and Patrick, 2016). Moreover, a review of over one million Medicaid enrollees showed that 21.6% of pregnant women had an opioid prescription filled, and 2.5% received prescriptions for greater than 30 days for chronic pain (Yazdy et al., 2015; Krans and Patrick, 2016).

Clinical studies have demonstrated that perinatal exposure to opioids can lead to long-lasting consequences such as cognitive deficits and sensorimotor impairments (Mactier and Hamilton, 2020). Recent imaging studies have also reported low-term changes in neural functioning after opioid exposure (Radhakrishnan et al., 2022; Vishnubhotla et al., 2022). These studies, however, were primarily focused on heroin and methadone and may not represent the effects of high-potency synthetic opioids like fentanyl. The available preclinical investigations of prenatal and postnatal exposure to fentanyl, however, suggest even brief can have seemingly permanent effects on mu-opioid functioning, affective behavior, and sensorimotor systems (Thornton and Smith, 1998; Medeiros et al., 2011; Alipio et al., 2021a,b; Rêgo et al., 2022).

The goal of the current investigation was to extend our understanding of fentanyl exposure during pregnancy and early development by assessing the effect of fentanyl administration from postnatal day (PD) 4 to PD 9 in late adolescent and young adult rats. Specifically, we examined morphine and U50, 488 thermal antinociception and oral fentanyl self-administration in both male and female rats. The fentanyl exposure time frame was chosen as it is roughly analogous to the last trimester of human pregnancy in terms of brain development (Schmitt and Barrow, 2022). While the third trimester only is not the most common exposure period for human women, it does represent a significant percentage of women who are prescribed opioids for pain during pregnancy (Cook, 2022).

## Materials and methods

### Animals

Male and female rats (*N* = 488) of Sprague–Dawley descent, born and raised at CSUSB, were used in both experiments. The day of parturition was considered PD 0, and litters were culled to a maximum of 10 rat pups at 3 days of age. Pups were kept with the dam until PD 23, at which time they were weaned and placed in group cages with same-sex litter mates. Only one rat from each litter was placed into a particular group. The colony room was maintained at 22–24°C and kept under a 12-h light/dark cycle. This study was approved by the Institutional Animal Care and Use Committee at California State University, San Bernardino. All studies were carried out in accordance with the “Guide for the Care and Use of Mammals in Neuroscience and Behavioral Research” (National Research Council, 2010).

### Drugs

Fentanyl citrate salt, morphine sulfate salt, and (±)-trans-U50-488 methanesulfonate salt were purchased from Sigma-Aldrich. Fentanyl used for the neonatal pre-exposure was mixed in saline, given at a volume of 2.5 ml/kg, and injected subcutaneously (sc). Oral fentanyl was dissolved in distilled water or a sucrose solution. Morphine and U50-488 were mixed in saline and injected subcutaneously (sc) at a volume of 1 ml/kg. Drug doses were expressed in the forms listed above.

### *In vivo* drug treatment and group assignment

On postnatal day (PD) 2, rats were sexed and assigned to fentanyl pre-treatment groups. In all cases, an equal number of male and female rat pups were allocated to each pre-treatment group. Drug assignments were coded, so experimenters were unaware of the drug dose administered. Starting at PD 4, male and female rats were weighed and injected with fentanyl (0, 10, or 100 μg/kg, sc). Fentanyl was given in two injections 6 h apart for 6 consecutive days.

### Hot plate test

On PD 60, rats were habituated to the hot plate apparatus (Model 38D, Hot plate analgesia meter, IITC Inc., Woodland Hills, CA, USA). Habituation consisted of placing the rat on the unheated hot plate for 2 min. On the next day, rats were placed on the heated hot plate (54.0°C, ± 0.1°C), and latency to lick a hind paw or attempt to jump out of the chamber was measured. This procedure was repeated three times, with a 20-min interval between each trial. After these baseline trials, rats were injected with morphine (0, 2.5, 5, or 10 mg/kg, sc) or U50, 488 (0, 5, 10, and 20 mg/kg, sc) and returned to their home cage for 20 min. Rats were then tested three additional times with a 20-min interval between each trial. If no response was made, rats were removed from the hotplate for 30 s to avoid tissue damage.

### Oral sucrose and fentanyl administration

#### Nose-poke training

Starting on PD 40, rats were pre-exposed to a 2% sucrose solution for 2 h in their home cage. Rats were then water deprived for 16 h. On the following day (i.e., PD 38), rats were placed in a self-administration chamber. Rats were allowed to nose poke for access to a 1% sucrose (w/v) solution on an FR1 schedule for 60 min each day until a criterion of >10 reinforces for 2 consecutive days was met. Nose-poke responses in the active hole resulted in the simultaneous presentation of a stimulus light and a sound cue (500 Hz, 10 dB above background) followed by a 30 s presentation of a liquid dropper that delivered 0.1 ml of the sucrose solution for 30 s. On nose-poke training days, water availability was restricted to 2 h day to accelerate the acquisition of operant responding. Rats returned to an ad-lib water schedule once the nose-poke criterion was met. Rats that fail to meet the training criteria were excluded from the study.

#### Self-administration

Once the criterion was met, fentanyl fade in and sucrose fade out began. Each nose-poke response in the active hole resulted in the simultaneous presentation of a stimulus light and a sound cue (500 Hz, 10 dB above background) and a 30 s presentation of a liquid dropper that delivered 0.1 ml of liquid solution. After each liquid dropper presentation, the active nose-poke hole became inactive for 20 s, which was indicated by the absence of the house light. Sessions 1–3 presented liquid solutions on a fixed ratio one (FR1) schedule where access to the liquid dropper occurred after every nose poke and sessions 4–5 presented liquid solutions on an FR2 schedule (i.e., two nose pokes were required before the presentation of the liquid dropper). Session 1 served as a baseline, where 1% sucrose solution was presented alone. In session 2, fentanyl (1 mg/L) was introduced into the sucrose solution. This dose of fentanyl is lower than that has been used in past studies (Shaham et al., 1993; Thornton et al., 2000) but was chosen to determine whether our early fentanyl exposure would increase the reinforcing value of fentanyl in adolescence. In session 3, sucrose fade out began with 0.5% sucrose presented in the liquid solution. In session 4, 0.25% sucrose was presented in the liquid solution. In session 5, no sucrose was present in the liquid solution. Sessions 1–4 were repeated until the criterion of >10 reinforces for 2 consecutive days is met. Session 5 was repeated for 7 days.

### Statistical analysis

Body weight during the pre-treatment period was analyzed by 2 × 3 × 6 (sex × pre-treatment condition × pre-treatment day) repeated measures ANOVA. Adult weight was analyzed by 2 × 3 (sex × pre-treatment condition) ANOVA. The three baseline paw-lick trials were averaged and analyzed by 2 × 3 (sex × pre-treatment condition) ANOVAs. Data from the postdrug paw-lick assessment were also averaged over the three test trials but were analyzed by Kruskal–Wallis and Mann–Whitney tests because these data did not meet the normality of distribution or homogeneity of variance assumptions. The total nose pokes and days to criterion were analyzed for sucrose training using 2 × 3 (sex × pre-treatment condition) ANOVAs. Total nose pokes and days to criterion for acquisition training phases 1–4 were analyzed using 2 × 3 × 4 (sex × pre-treatment condition × training phase) repeated measures ANOVAs. Total nose pokes for the seven-phase 5 testing days (fentanyl-only sessions) were analyzed using 2 × 3 × 7 (sex × pre-treatment condition × day) repeated measures ANOVAs. Significant higher-order interactions were analyzed using lower-order ANOVAs. *Post hoc* analysis of data was made using Tukey’s tests (*p* < 0.05). Effect sizes were reported as partial eta squared (η*_*p*_*^2^) and categorized based on the following scale: η*_*p*_*^2^ ≤ 0.03 (small effect), η*_*p*_*^2^ > 0.03 and ≤0.10 (medium effect), and η*_*p*_*^2^ > 0.10 (large effect) (Labots et al., 2016).

## Results

### Body weight

**FIGURE 1:**
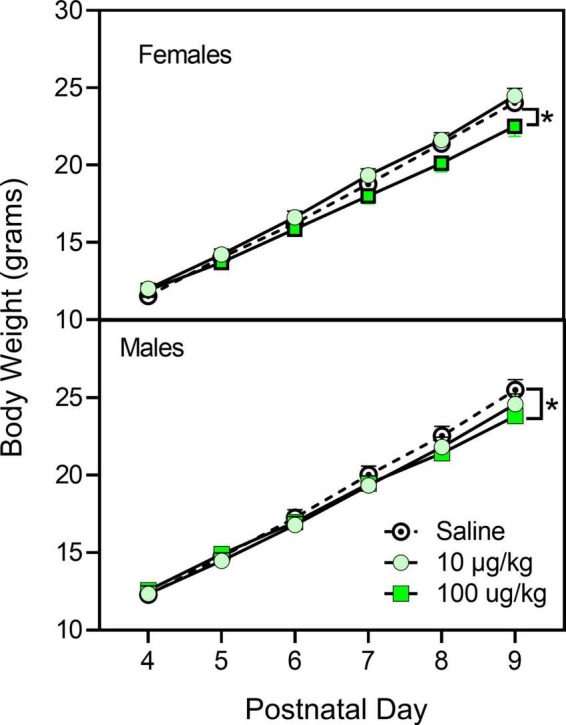
Mean (±SEM) body weight of male and female rats (*n* = 8–9/sex) treated with saline or fentanyl (10 or 100 μg/kg) from PD 4 to PD 9. *Significant difference between vehicle- and 100 μg/kg fentanyl-treated rats of the same sex.

During the injection period (i.e., PD 4–9), male rat pups were slightly larger than female rat pups (Figure 1) [sex main effect, *F*_(1,407)_ = 8.828, *p* = 0.003, η*_*p*_^2^* = 0.021]. Weight increased progressively for all pups on the injection days, and pre-treatment with fentanyl (100 μg/kg) decreased weight in comparison with saline controls for both males and females on the last pre-treatment day (PD 9) [day main effect, *F*_(2,1713)_ = 10,486.243, *p* < 0.001, η*_*p*_^2^* = 0.963; day × pre-treatment condition interaction, *F*_(4,1713)_ = 6.99, *p* < 0.001, η*_*p*_^2^* = 0.064]. Weight for males and females did not differ from controls at the time of testing (data not shown).

### Morphine- and U50-488-induced thermal antinociception

**FIGURE 2:**
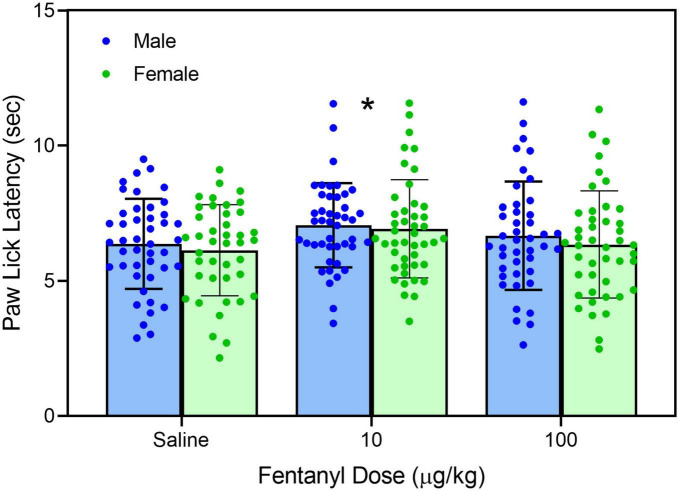
Mean (+SE) baseline paw-lick latencies of PD 60 male and female rats (*n* = 8–9/sex). Rats were pre-treated with fentanyl (10 or 100 μg/kg) or saline from PD 4 to PD 9. *Significantly different from rats in the saline-pre-treatment group.

**FIGURE 3:**
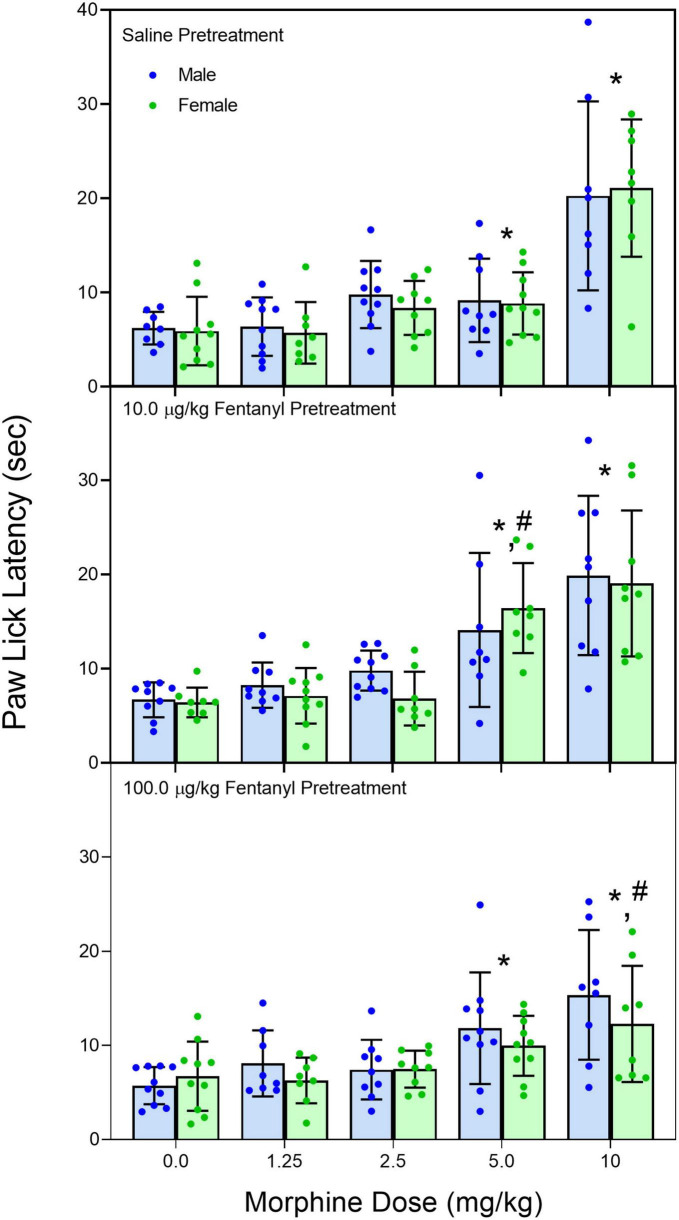
Mean (+SE) paw-lick latencies of PD 60 male and female rats (*n* = 8–9/sex). Rats were pre-treated with fentanyl (10 or 100 μg/kg) or saline from PD 4 to PD 9 and injected with morphine (0, 1.25, 2.5, 5.0, or 10 mg/kg, sc) on PD 60. *Significantly different from 0 mg/kg morphine. ^#^Significantly different from saline-pre-treated rats in the same morphine drug condition.

**FIGURE 4:**
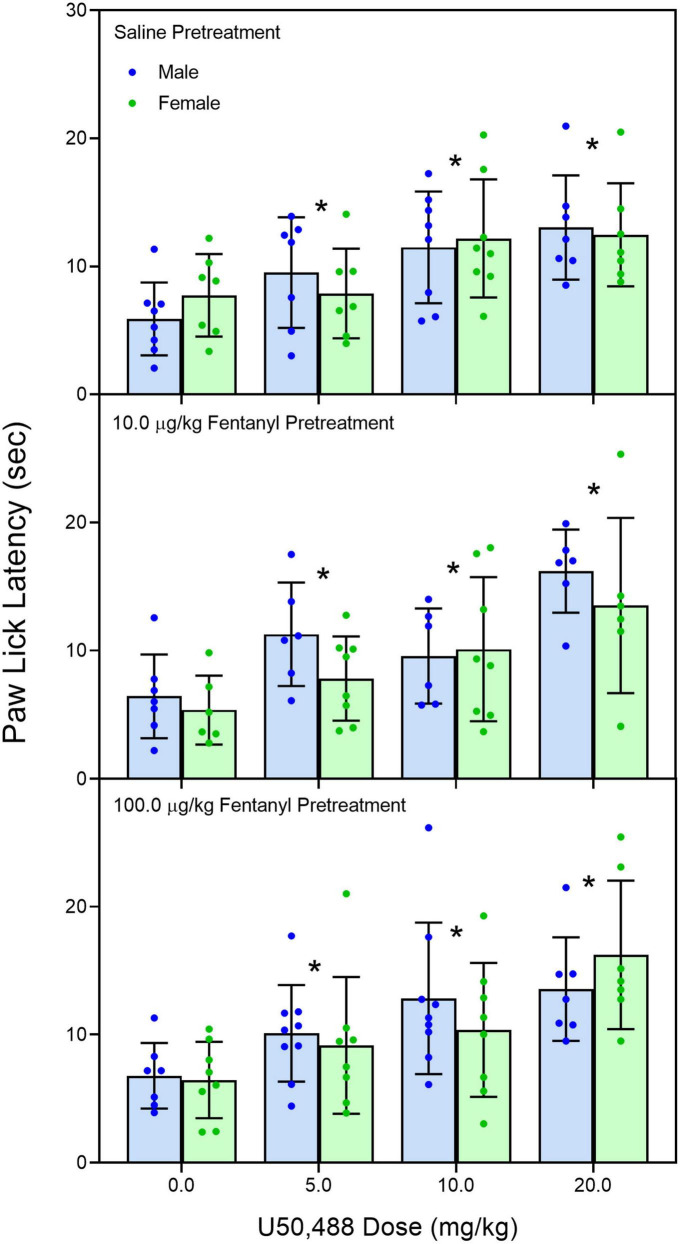
Mean (+SE) baseline paw-lick latencies of PD 60 male and female rats (*n* = 7–8/sex). Rats were pre-treated with fentanyl (10 or 100 μg/kg) or saline from PD 4 to PD 9 and injected with U50,488 (0, 5.0, 10, or 20 mg/kg, sc) on PD 60. *Significantly different from 0 mg/kg morphine.

Early neonatal treatment with fentanyl (10 μg/kg) altered the baseline responses on the hotplate for both male and female rats on PD 60 (Figure 2) [fentanyl pre-treatment main effect, *F*_(2,256)_ = 3.388, *p* < 0.035; η*_*p*_^2^* = 0.026; Tukey’s test, *p* < 0.05]. As expected, treatment with morphine (2.5, 5.0, and 10 mg/kg) increased paw-lick latencies regardless of the pre-treatment group (Figure 3) [morphine post-treatment, *H*(4) = 96.156, *p* < 0.001; η*_*p*_^2^* = 0.368; pairwise comparison with Bonferroni correction, *p* < 0.05]. The antinociceptive effects of morphine were altered by the early fentanyl treatment (Figure 3). Specifically, rats pre-treated with fentanyl (10 μg/kg) and tested with 5 mg/kg of morphine had greater paw-lick latencies than saline-treated rats, [5 mg/kg morphine × fentanyl groups, *H*(2) = 8.583, *p* < 0.014, η*_*p*_*^2^ = 0.170, Mann–Whitney U, *p* = 0.033]. In addition, rats treated with 100 μg fentanyl and tested with 10 mg/kg morphine had shorter paw-lick latencies than saline controls [10 mg/kg morphine × fentanyl groups, *H*(2) = 6.372, *p* < 0.041, η*_*p*_*^2^ = 0.132; Mann–Whitney U, *p* = 0.0235]. Sex did not alter morphine-induced thermal antinociception. U50-488 (5, 10, and 20 mg/kg) also increased paw-lick latencies (Figure 4) [U50, 488 post-treatment, *H*(3) = 48.371, *p* < 0.001; η*_*p*_*^2^ = 0.281; pairwise comparison with Bonferroni correction, *p* < 0.05], but the effect of the kappa agonist was not altered by fentanyl pre-treatment (Figure 4).

### Oral-fentanyl self-administration

#### Sucrose training

Active nose pokes, inactive nose pokes, sucrose solution consumed, or days to criterion were not altered by early fentanyl treatment. Only three rats failed to reach our criterion on the sucrose training procedure. Male rats, however, did have greater numbers of active and inactive lever presses [sex main effect *F*_(1,54)_ = 5.779, *p* = 0.02, η*_*p*_*^2^ = 0.097; *F*_(1,54)_ = 4.343, *p* = 0.042, η*_*p*_*^2^ = 0.074, respectively].

#### Acquisition of oral-fentanyl self-administration—Training phase

**FIGURE 5:**
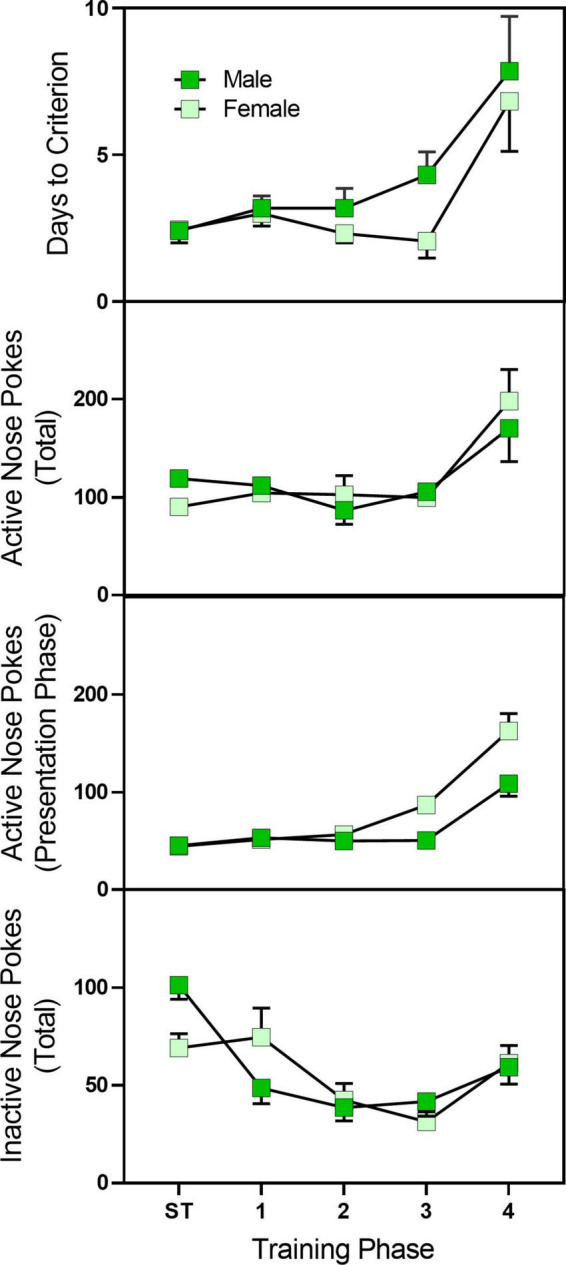
Mean (±SEM) active nose pokes, active nose pokes during the stimulus presentation, and inactive nose pokes in late adolescent male and female rats (*n* = 23–27/sex) during sucrose training and acquisition training for sucrose/fentanyl-rewarded nose pokes. Rats were pre-treated with fentanyl (10 or 100 μg/kg) or saline from PD 4 to PD 9 and started sucrose training on PD 40. Rats first were trained to nose poke for a sucrose solution and then underwent a four-phase sucrose fade out/fentanyl fade in procedure. ST, sucrose training.

Pre-treatment with fentanyl did not alter active nose pokes, inactive nose pokes, or days to criterion (data not shown). However, female rats had a slightly greater number of active nose pokes during the stimulus presentation as compared to male rats (Figure 5) [sex main effects *F*_(1,39)_ = 5.072, *p* = 0.030, η*_*p*_*^2^ = 0.079]. Days to criterion were not affected by sex. In total, 11 rats (seven male rats and four female rats) failed to complete the training protocol.

#### Acquisition of oral-fentanyl self-administration—Test phase

**FIGURE 6:**
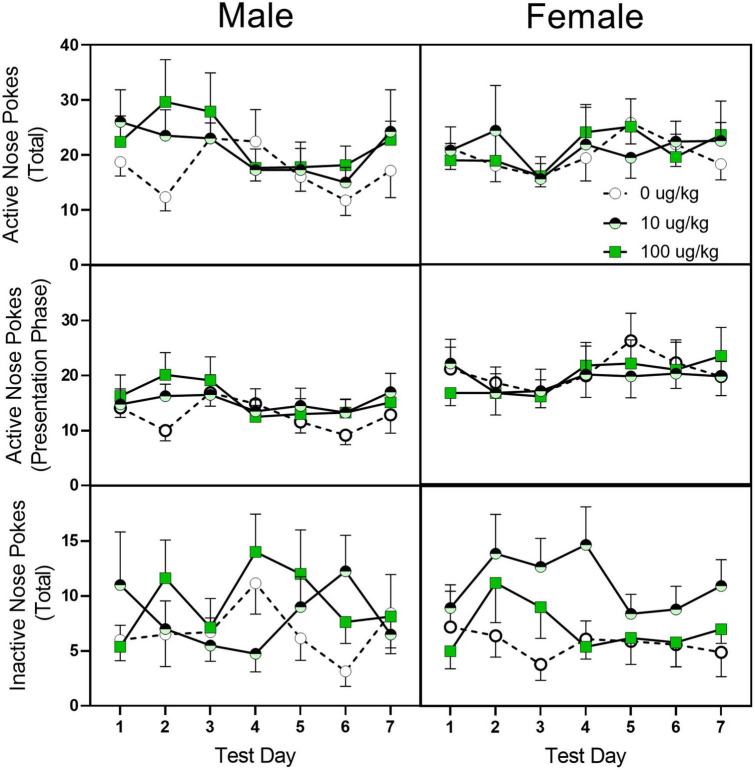
Mean (±SEM) active nose pokes, active nose pokes during the stimulus presentation, and inactive nose pokes in late adolescent male and female rats (*n* = 6–8/sex) during the seven fentanyl-only acquisition test days. Rats were pre-treated with fentanyl (10 or 100 μg/kg) or saline from PD 4 to PD 9 and started sucrose training on PD 40. After reaching the criterion for sucrose-rewarded nose pokes, rats underwent a four-phase sucrose fade out/fentanyl fade in procedure and then were assessed for 7 days for fentanyl-only reinforcement.

Similar to the training phase, fentanyl pre-treatment did not alter active or inactive nose pokes during the 7-day test days (see Figure 6). Female rats, however, did have a greater number of active nose pokes, active nose pokes during the stimulus presentation, and inactive nose pokes [sex main effect *F*_(1,40)_ = 4.295, *p* = 045, η*_*p*_*^2^ = 0.202; *F*_(1,40)_ = 6.253, *p* = 0.017, η*_*p*_*^2^ = 0.266; *F*_(1,40)_ = 5.746, *p* = 0.021, η*_*p*_*^2^ = 0.226, respectively].

## Discussion

In the present study, brief neonatal fentanyl exposure had long-term effects on opioid-mediated behavior in adolescent and young adult rats. Specifically, we found that fentanyl administered from PD 4 to PD 9 caused long-term changes in morphine-induced thermal antinociception of young adult rats. While we failed to find other studies that used the same injection period as in our study, the change in morphine thermal antinociception we reported is consistent with another study that did fentanyl infusions from PD 14 to PD 17 (Thornton and Smith, 1998). Moreover, these results are in agreement with prenatal opioid exposure investigations showing both an increase and decrease in response to morphine in adult offspring depending on injection interval (O’Callaghan and Holtzman, 1976, 1977; Kirby et al., 1982). Curiously, early fentanyl exposure did not alter nose-poke response to oral fentanyl in late adolescence.

The changes we found in morphine thermal antinociception were dose-dependent because exposure to a low dose (10 μg/kg) resulted in an augmented response to 5 mg/kg of morphine while pre-treatment with (100 μg/kg) caused an attenuated response to 10 mg/kg morphine. Decreased morphine sensitivity has been previously reported after early postnatal fentanyl exposure (Thornton and Smith, 1998) and is often found after prenatal or early postnatal exposure to other mu-opioids (O’Callaghan and Holtzman, 1976; Timár et al., 2010). In these studies, the decrease in morphine sensitivity is seen after an early opioid exposure protocol that induces opioid dependence (Thornton and Smith, 1998); while the current study did not directly assess fentanyl dependence, our drug protocol (two 50 μg/kg injections; 6 h apart for 6 days) is similar to other protocols that have induced tolerance in young rats (Laferrière et al., 2005). The decrease in responsivity to morphine is likely due to long-lasting changes in mu-opioid receptors (i.e., receptor density, affinity, or G-protein coupling) as prior studies have demonstrated that morphine pharmacokinetics are not changed after early opioid exposure (O’Callaghan and Holtzman, 1976). Moreover, the current study showed that kappa receptors were unchanged as U50, 488 thermal antinociception was not affected by fentanyl pre-treatment.

The increased analgesic response to morphine after pre-exposure to the low dose of fentanyl was not anticipated based on most of the literature on early life exposure to mu-opioid agonists because these studies showed a decreased response to later opiate exposure or no change (O’Callaghan and Holtzman, 1976; Thornton and Smith, 1998; Timár et al., 2010). There was one published report where the morphine had a greater antinociceptive response after early fentanyl treatment, but that report used a protocol that should have induced tolerance and the nociceptive task (tail flick) measured spinal mediated thermal antinociception (Kirby et al., 1982). While again we did not assess the development of physical dependence on fentanyl, our protocol (two 5 μg/kg injections; 6 h apart for 6 days) was probably not sufficient based on other published reports. It is possible that instead of causing long-term desensitization of mu receptors, the low-dose fentanyl treatment upregulated or increased receptor sensitivity. This is of course speculative but not improbable and may be reflective of changes to mu receptor/G-protein coupling or intrinsic activity (Windh et al., 1995; Thornton et al., 1998).

Based on the thermal antinociception study, we expected that our fentanyl pre-treatment would alter oral fentanyl self-administration. However, we found almost no effect of early postnatal fentanyl pre-treatment on fentanyl-reinforced nose pokes. The one exception to this was a slight increase in inactive nose pokes in female rats treated with the low dose of fentanyl (10 μg/kg/day). Our failure to find enhanced fentanyl reward, however was consistent with another early fentanyl pre-treatment study that found that rat pups made dependent on fentanyl did not respond differently than saline-treated rats for oral fentanyl (Thornton et al., 2000) and a prenatal morphine paper where morphine- and saline-exposed rats did not differ on morphine-conditioned place preference task or morphine self-administration [Riley and Vathy, 2006; but see Timár et al. (2010)]. It is not known why morphine thermal antinociception was altered but fentanyl-rewarded responding was not, but it is possible that the difference is a result of morphine being a less efficacious agonist as compared to fentanyl (Grecksch et al., 2011; Cornelissen et al., 2018). It is possible that the desensitization caused by fentanyl was sufficient to blunt the morphine response but not the response to fentanyl. Alternatively, it is possible that the subset of receptors responsible for the antinociceptive effects was affected more by the early fentanyl treatment than the receptors necessary for reward. This explanation would agree with a study showing that early morphine treatment had opposite effects on morphine thermal antinociception and morphine-conditioned place preference (Timár et al., 2010). Finally, it is also possible that our procedure was not optimal for assessing differences in the rewarding properties of fentanyl. For example, we used a dose of fentanyl that was below the concentration typically used for self-administration, and it is conceivable that a higher concentration may have resulted in a more marked difference in response (Shaham et al., 1993; Thornton et al., 2000). In addition, while oral operant self-administration has proven to be effective at predicting the reinforcing value of a drug [see Wilson et al. (1997)], a two-bottle choice procedure may have given a better measure of drug preference.

Unlike the fentanyl pre-treatment, sex did alter nose-poke behavior on the oral fentanyl self-administration task because female rats consistently responded at higher rates for fentanyl. This finding is in agreement with other preclinical studies showing that female rats had a greater intake of intravenous fentanyl (Malone et al., 2021; Towers et al., 2022), heroin (George et al., 2021), and oral oxycodone (Fulenwider et al., 2020). Clinically, the role of sex in fentanyl and other opioid use is more complicated. Men have higher rates of opioid use disorder and opioid-related deaths as compared to women, but women show a stronger craving for drug cues, develop opioid use disorder faster than males, and are more likely to be prescribed opioid analgesics for pain management than men (Back et al., 2011; Wightman et al., 2021; Martin et al., 2022; Romanescu et al., 2022).

In summary, we found that exposure to fentanyl during the early neonatal period has long-lasting consequences on morphine thermal antinociception but did not enhance the rewarding effects of fentanyl in late adolescence. While our exposure period modeling third-trimester exposure only is not typical of human exposure, it does show even brief exposure to fentanyl can have a major impact on later functioning. Importantly, our findings also indicate that adolescent females are more vulnerable to fentanyl use than males.

## Data availability statement

The raw data supporting the conclusions of this article will be made available by the authors, without undue reservation.

## Ethics statement

This animal study was reviewed and approved by Institutional Animal Care and Use Committee, California State University, San Bernardino.

## Author contributions

CC contributed to the conception and design of the study, performed the statistical analyses, and wrote the first draft. JT, GP, JR, JB, and CG conducted the morphine thermal antinociception and the self-administration experiments. JT, DL, and DG conducted the kappa thermal antinociception experiment. All authors contributed to the manuscript revision and approved the submitted version.
